# Linear combination of one-step predictive information with an external reward in an episodic policy gradient setting: a critical analysis

**DOI:** 10.3389/fpsyg.2013.00801

**Published:** 2013-11-04

**Authors:** Keyan Zahedi, Georg Martius, Nihat Ay

**Affiliations:** ^1^Max Planck Institute for Mathematics in the SciencesLeipzig, Germany; ^2^Santa Fe InstituteSanta Fe, NM, USA

**Keywords:** information-driven self-organization, predictive information, reinforcement learning, embodied artificial intelligence, embodied machine learning

## Abstract

One of the main challenges in the field of embodied artificial intelligence is the open-ended autonomous learning of complex behaviors. Our approach is to use task-independent, information-driven intrinsic motivation(s) to support task-dependent learning. The work presented here is a preliminary step in which we investigate the predictive information (the mutual information of the past and future of the sensor stream) as an intrinsic drive, ideally supporting any kind of task acquisition. Previous experiments have shown that the predictive information (PI) is a good candidate to support autonomous, open-ended learning of complex behaviors, because a maximization of the PI corresponds to an exploration of morphology- and environment-dependent behavioral regularities. The idea is that these regularities can then be exploited in order to solve any given task. Three different experiments are presented and their results lead to the conclusion that the linear combination of the one-step PI with an external reward function is not generally recommended in an episodic policy gradient setting. Only for hard tasks a great speed-up can be achieved at the cost of an asymptotic performance lost.

## 1. Introduction

One of the main challenges in the field of embodied artificial intelligence (EAI) is the open-ended autonomous learning of complex behaviors. Our approach is to use task-independent, information-driven intrinsic motivation to support task-dependent learning in the context of reinforcement learning (RL) and EAI. The work presented here is a first step into this direction. RL is of growing importance in the field of EAI, mainly for two reasons. First, it allows to learn the behaviors of high-dimensional and complex systems with simple objective functions. Second, it has a well-established theoretical (Sutton and Barto, 1998; Bellman, 2003) and biological foundation (Dayan and Balleine, 2002). In the context of EAI, where the agent has a morphology and is situated in an environment, the concepts of the agent's intrinsic and extrinsic perspective rise naturally. As a direct consequence, several questions about intrinsic and extrinsic reward functions, denoted by IRF and ERF, follow from the EAI's point of view. The questions that are of interest to us are; what distinguishes an IRF from an ERF, what is a good candidate for a first principled IRF, and finally, how should IRFs and ERFs be combined?

The first question, of how to distinguish between IRF and ERF is addressed in the second section of this work, which starts with the conceptual framework of the sensorimotor loop and its representation as a causal graph. This leads to a natural distinction of variables that are intrinsic and extrinsic to the agent. We define an IRF that models an internal drive or motivation as a task-independent function which operates on the agent's intrinsic variables only. In general, an ERF is a task-dependent function that may operate on intrinsic and extrinsic variables.

The main focus of this work is the second question, which deals with finding a first principled IRF. We propose the predictive information (PI) (Bialek et al., 2001) for the following reasons. Information-driven self-organization, by the means of maximizing the one-step approximation of the PI has proved to produce a coordinated behavior among physically coupled but otherwise independent agents (Ay et al., 2008; Zahedi et al., 2010). The reason is that the PI inherently addresses two important issues of self-organized adaptation, as the following equation shows: *I*(*S*_*t*_; *S*_*t* + 1_) = *H*(*S*_*t* + 1_) − *H*(*S*_*t* + 1_|*S*_*t*_), where *S*_*t*_ is the vector of intrinsically accessible sensor values at time *t*. The first term leads to a diversity of the behavior, as every possible sensor state must be visited with equal probability. The second term ensures that the behavior is compliant with the constraints given by the environment and the morphology, as the behavior must be predictable. This means that an agent maximizing the PI explores behavioral regularities, which can then be exploited to solve a task. In a differently motivated work, namely to obtain purely self-organizing behavior, a time-local version of the PI was successfully used to drive the learning process of a robot controller (Martius et al., 2013). A similar learning rule was obtained from the principle of Homeokinesis (Der and Martius, 2012). In both cases a gradient information was derived to pursue local optimization. For the integration of external goals a set of methods have been proposed by (Martius and Herrmann, 2012), which, however, cannot deal with the standard reinforcement setting of arbitrary time-delayed rewards that we study here. Prokopenko et al. (2006) used the PI, estimated on the spatio-temporal phase-space of an embodied system, as part of fitness function in an artificial evolution setting. It was shown that the resulting locomotion behavior of a snake-bot was more robust, compared to the setting, in which only the traveled distance determined the fitness.

The third question, which deals with how to combine the IRF and ERF, is in the focus of the ongoing research that was briefly described above and of which this publication is a first step. As the PI maximization is considered to be an exploration of behavioral regularities, it would be natural to exchange the exploration method of a RL algorithm by a gradient on the PI. The work presented here is a preliminary step in which we concentrate on the effect of the PI in a RL context to understand for which type of learning problems it is beneficial and in which it might not be. Therefore, we chose a linear combination of IRF and ERF in an episodic RL setting to evaluate the PI as an IRF in different experiments. Combining an IRF and an ERF in this way is justified as ERFs are often linear combinations of different terms, such as one term for fast locomotion and another for low energy consumption. Nevertheless, the results of the experiments presented in this work show that the one-step PI should not be combined in this way with an ERF in an episodic policy gradient setting.

We are not the first to address the question of IRF and ERF in the context of RL and EAI. This idea goes back to the pioneering work of Schmidhuber (1990) and is also in the focus of more recent work (Kaplan and Oudeyer, 2004; Schmidhuber, 2006; Oudeyer et al., 2007) which are based on the prediction progress and Barto et al. (2004), who considers the prediction error. In Storck et al. (1995); Yi et al. (2011) an intrinsic reward for information gain was proposed (KL-divergence between subsequent models), which results in their experiments in a state-entropy maximization. A different approach (Little and Sommer, 2013) uses a greedy policy on the predicted information gain of the world model to select the next action of an agent. However, only discrete state/action spaces have been considered in both approaches. A similar work (Cuccu et al., 2011) uses compression quality as the intrinsic motivation, which was particularly beneficial because it performed a reduction of the high-dimensional visual input space. In comparison to our work only one experiment (comparable to the self-rescue task below) with a one-dimensional action-space was used without considering asymptotic performance, which is where we found most problems.

This paper investigates continuous space high-dimensional control problems where random exploration becomes difficult. The PI, measured on the sensor values, accompanies (and might eventually replace) the exploration of a RL method such that the policy adaptations are conducted compliant to the morphology and environment. The actual embodiment is taken into account, without modeling it explicitly in the learning process.

The work is organized in the following way. The next section gives an overview of the methods, beginning with the sensorimotor loop and its causal representation. This is then followed by a presentation of the PI and the episodic RL method PGPE (Sehnke et al., 2010). The third section describes the results received by applying the methods to three experiments, and the last section closes with a discussion.

## 2. Methods

This section describes the methods used in this work. It begins with the conceptual framework of the sensorimotor loop. This is then followed by a discussion of the PI and entropy, which both are used as IRF in all presented experiments. Finally, the RL algorithm utilized in this work is introduced as far as it is required to understand how the results were obtained.

### 2.1. Embodied agents and the sensorimotor loop

**Figure 1:**
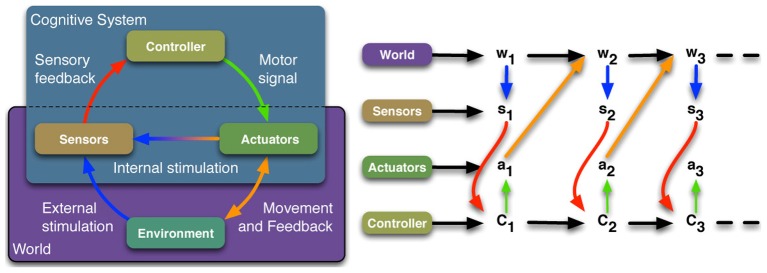
**The sensorimotor loop. Left:** Schematic diagram of a cognitive system with its interaction with the world. **Right:** Corresponding causal graph.

There are three main reasons why we prefer to experiment with embodied agents (EA). First, *scalability*: EA are high-dimensional systems which live in a continuous world. Hence, the algorithms face the curse of dimensionality if they are evaluated on different EAs. Second, *validation*: we are interested in understanding natural cognitive systems by the means of building artificial agents (Brooks, 1991). Using EA ensures that the models are validated against the same (or similar) physical constraints that natural systems are exposed to. Third, *guidance:* there is good evidence that the constraints posed by the morphology and environment can be used to reduce the required controller complexity, and hence, reduce the size of the search space for a learning algorithm (Zahedi et al., 2010; Pfeifer and Bongard, 2006). Consequently, understanding the interplay between the body, brain and environment, also called the sensorimotor loop (SML, see Figure 1), is a general focus of our work. The next paragraph will introduce the general concept of the SML and discuss its representation as a causal graph.

A cognitive system consists of a brain or controller that sends signals to the system's actuators, which then affect the system's environment. We prefer the notion of the system's *Umwelt* (von Uexkuell, 1934; Clark, 1996; Zahedi et al., 2010; Zahedi and Ay, 2013), which is the part of the system's environment that can be affected by the system, and which itself affects the system. The state of the actuators and the *Umwelt* are not directly accessible to the cognitive system, but the loop is closed as information about both, the *Umwelt* and the actuators are provided to the controller by the system's sensors. In addition to this general concept, which is widely used in the EAI community (see e.g., Pfeifer et al., 2007), we introduce the notion of *world* to the sensorimotor loop, and by that we mean the system's morphology and the system's *Umwelt*. We can now distinguish between the agent's intrinsic and extrinsic perspective in this context. The world is everything that is extrinsic from the perspective of the cognitive system, whereas the controller, sensor and actuator signals are intrinsic to the system.

The distinction between intrinsic and extrinsic is also captured in the representation of the sensorimotor loop as a causal or Bayesian graph (see Figure 1, right-hand side). The random variables *C, A, W*, and *S* refer to the controller state, actuator signals, world and sensor signals, and the directed edges reflect causal dependencies between the random variables (see Klyubin et al., 2004; Ay and Polani, 2008; Zahedi et al., 2010). Everything that is extrinsic to the system is captured in the variable *W*, whereas *S, C*, and *A* are intrinsic to the system.

In this context, we distinguish between internal and external reward function (IRF, ERF) in the following way. An ERF may access any variable, especially those that are not available to an agent by its sensors, i.e., anything that we summarized as the world state *W*. An IRF may access intrinsically available information only (*S*_*t*_, *A*_*t*_, *C*_*t*_, see Figure 1). We are interested in first principled model of an intrinsic motivation, i.e., a model that requires as few assumptions as possible. The idea is that IRF should not depend on a specific task but rather be a task-independent internal driving force, which supports any task-dependent learning. This is why we refer to it as task-independent internal motivation or drive. This closes the discussion of embodied agents and their formalization in terms of the sensorimotor loop. The next section describes the information-theoretic measures that are used in the remainder of this work.

### 2.2. Predictive information

The predictive information (PI) (Bialek et al., 2001), which is also known as excess entropy (Crutchfield and Young, 1989) and effective measure complexity (Grassberger, 1986) is defined as the mutual information of the entire past and future of the sensor data stream:

(1) $$I_{\text{pred}}(S):=I(S_{p};S_{f})$$

where *S*_*p*_ = {*S*_1_, *S*_2_, …, *S*_*t*_} is the entire past of the system's sensor data at some time *t* ∈ ℕ and *S*_*f*_ = {*S*_*t* + 1_, *S*_*t* + 2_,…} its entire future. The PI captures how much information the past carries about the future. Unfortunately, it cannot be calculated for most applications because of technical reasons. Hence, we use the one-step PI, which is given by

(2) $$\begin{array}{l} I_{\text{pred}}^{*}(S):=I(S_{t\;+\;1};S_{t})\\ \;=\underset{\text{diversity}}{\underset{︸}{H(S_{t\;+\;1})}}\underset{\text{compliance}}{\underset{︸}{-H(S_{t\;+\;1}|S_{t})}}, \end{array}$$

which was previously investigated in the context of EAI (Ay et al., 2008) and as a first principle learning rule (Zahedi et al., 2010; Martius et al., 2013). A different motivation for the PI is based on maximizing the mutual information of an intention state ${\tilde{S}}_{t}$, which is internally generated by the agent, and the next sensor state *S*_*t* + 1_ (Ay and Zahedi, 2013). The Equation (2) displays how maximizing the PI affects the behavior of a system. The first term in Equation (2) leads to a maximization of the entropy over the sensor states. This means that the agent has to explore its world in order to sense every state with equal probability. The second term in Equation (2) states that the uncertainty of the next sensor state must be minimal if the current sensor state is known. This means that an agent has to choose actions which lead to predictable next sensor states. This can be rephrased in the following way. Maximizing the entropy *H*(*S*_*t* + 1_) increases the diversity of the behavior whereas minimizing the conditional entropy −*H*(*S*_*t* + 1_|*S*_*t*_) increases the compliance of the behavior. The result is a system that explores its sensors space to find as many regularities in its behavior as possible.

For completeness we will also maximize the entropy *H*(*S*_*t*_) only and compare the results to the maximization of the PI. This concludes the presentation of the PI (and entropy) as a model for a task-independent internal motivation in the context of RL. The next section presents the utilized RL algorithm.

### 2.3. Policy gradients with parameter-based exploration (PGPE)

We chose an episodic RL method named PGPE (Sehnke et al., 2010) to investigate the effect of the PI as an IRF, because it is not restricted to a specific class of policies. Any policy, which can be represented by a vector μ ∈ ℝ^*n*^ with fixed length *n* ∈ ℕ^+^ can be optimized by this method. In the work presented here, we use it to learn the synaptic strengths and bias values of neural networks with fixed structures only. Nevertheless, we can apply the framework to other parametrizations, in particular to stochastic policies, which is why PGPE attracted our attention for ongoing the project in which this work is embedded.

The algorithm can be summarized in the following way (for details, see (Sehnke et al., 2010)). In each *roll-out* or episode, two policy instances are drawn from μ by adding and subtracting a random vector ϵ ~ 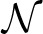
(0, σ) to it. The resulting two policy parametrizations Θ^+^ = μ + ϵ and Θ^−^ = μ − ϵ are then evaluated and their final rewards *r*^+^, *r*^−^ are used to determine the modifications on μ and σ according to the following equations

(3) $$m^{n}=\max(m^{n-1},r^{+,n},r^{-,n})$$

(4) $$b^{n}=(1-\delta )b^{n-1}+\delta \sum_{n}\frac{r^{+,n}+r^{-,n}}{2}$$

(5) $$\Delta \mu_{i}=\frac{\alpha \epsilon_{i}(r^{+}-r^{-})}{2m-r^{+}-r^{-}}$$

(6) $$\Delta \sigma_{i}=\frac{\alpha }{m-b}\left(\frac{r^{+}-r^{-}}{2}-b\right)\left(\frac{\epsilon^{2}-\sigma_{i}^{2}}{\sigma_{i}}\right).$$

Roll-outs can be repeated several times before a learning step is performed. Every learning step concludes a *batch*. PGPE requires an initial μ_init_, an initial σ_init_, a learning rate α, baseline *b*, baseline adaptation parameter δ, and an initialized maximal reward *m* = *m*_init_. We have set δ to the recommended value of 0.1, μ_init_ = 0, and we have achieved the best results in all experiments by setting *m*_init_ small enough that *m* is definitely overwritten in the first roll-out (see Equation (3)). The other parameters are evaluated in each experiment, such that the best results were achieved when no IRF was used and then fixed for the remaining experiments.

## 3. Results

This section presents three different experiments and their results. The first experiment is the cart-pole swing-up, a standard control theory problem that is also widely used in machine learning (Barto et al., 1983; Geva and Sitte, 1993; Doya, 2000; Pasemann et al., 1999). The cart-pole experiment is also chosen because balancing a pole minimizes the entropy, and hence, it contradicts the maximization of the PI. The second experiment is the learning of a locomotion behavior for a hexapod and it was chosen to demonstrate the effect of the PI maximization on a more common, well-structured experimental setting. By well-structured we mean that the controller, morphology, environment, and ERF are chosen such that they result in a good hexapod locomotion without any additional support by an IRF in only a few policy updates. The third experiment is designed to be challenging, as it combines a high-dimensional system, an unconventional control structure, an unsteady ERF with an unsteady environment. We believe that these three experiments span a broad range of possible applications for information-theoretic IRF in the context of episodic RL.

### 3.1. Cart-pole swing-up

The cart-pole swing-up experiment is ideal to investigate the effect of the PI on an episodic RL task, mainly for two reasons. First, the experiment is well-defined by a set of equations and parameters that are widely used in literature (Barto et al., 1983; Geva and Sitte, 1993; Doya, 2000; Pasemann et al., 1999). This ensures that the results are comparable and reproducible by others with little effort. Second, the successful execution of the task contradicts the maximization of the PI. The task is to balance the pole in the center of the environment, and hence, to minimize the entropy of the sensor states. The maximization of the PI demands a maximization of the entropy (see Equation 2). The remainder of this section first describes the experimental and controller setting and then closes with a discussion of the results.

**Figure 2:**
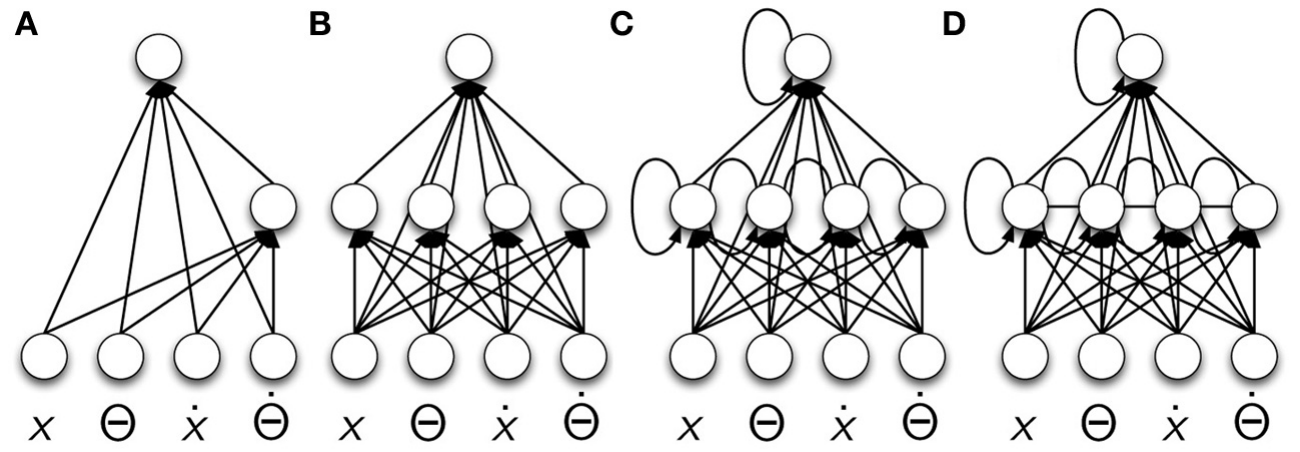
**Controller architectures for the cart-pole swing-up task**. The input neurons are bare buffer neurons whereas the hidden and output neurons have tanh transfer-function. **(A)** from Pasemann et al. (1999); **(B)** with 4 hidden neurons and fully connected; **(C,D)** recurrent variations without and with lateral connections.

The experiment was conducted by implementing the equations that can be found in (Barto et al., 1983; Geva and Sitte, 1993; Doya, 2000). The state of the cart-pole is given by *x*, *ẋ*, *ϑ*, $\dot{\vartheta }$, which are the position of the cart, the speed of the cart, the pole angle and the pole's angular velocity. The cart is controlled by a force *F* ∈ [−10*N*, 10*N*] that is applied to its center of mass. The four state variables and the force define the input and output configuration of our controllers for this task. The initial controller (see Figure 2A) was chosen from (Pasemann et al., 1999), where network structures were evolved for the same task. To ensure that the evolved structure is not especially unsuitable for RL, different variations were chosen for evaluation too (see Figures 2B–D). In this approach, the input neurons are simple buffer neurons, with the identity as transfer-function, whereas all other neurons use the hyperbolic tangent transfer-function.

The evaluation time was set to *T* = 2000 iterations, which corresponds to 20 seconds (c.f. Doya, 2000). Different values, starting from the values proposed in (Sehnke et al., 2010), for the learning rate α ∈ {0.1, 0.2, 0.5}, the initial variation σ_init_ ∈ {2, 5}, and the initial maximal reward *m*_init_ ∈ {−∞, 10, 100, 1000} were evaluated in experiments without applying an IRF to the learning of the task. The underlined values showed the best results, and hence, are chosen for presentation here. Each experiment consisted of *B* = 10000 batches, i.e., updates of μ and σ (see Equations 5 and 6) with two roll-outs each (i.e., four evaluated policies θ^+, −^_1, 2_). The results are obtained by conducting every experiment 100 times. To ensure comparability among the experiments with different parameters and controllers, the random number generator was initialized from a fixed set of 100 integer values for each experiment.

The presentation of the reward function is split into two parts. The first part handles the ERF, whereas the second part handles the IRF. We use the terms *intrinsic/internal* and *extrinsic/external* with respect to the agent's perspective, as discussed in the previous section (see Section 2.1). The controller has access to the full state of the system, and hence, the separation into internal and external is artificial in this case. Nevertheless, we keep this terminology for consistency, as the next experiments will reflect this distinction in a natural way. We denote IRF by *R*_in_ and ERF by *R*_ex_, where a super-script is added to distinguish between the different reward functions (PI and entropy).

The ERF for the cart-pole swing-up task is defined such that it is not a smooth gradient in the reward space, and therefore, does not directly guide the learning process. The controller is only rewarded if the pole is pointing upwards and the reward is scaled with the distance of the pole to the center of the environment, which is given by

(7) $$R_{\text{ex}}(t)\;:=\{\begin{array}{ll} 2-|x(t)| & \text{if }|\vartheta (t)|<5^{{}^{\circ}}\\ 0 & \text{otherwise}. \end{array}$$

The IRF is calculated at the end of each episode based on the recordings of the pole angles {*S*_*t*_ = ϑ(*t*)|*t* = 1, 2, …, *T*}. We use a discrete-valued computation of the PI, and hence, the data is binned prior to the calculation. All IRFs are normalized with respect to their theoretical upper bound of *I*(*S*_*t* + 1_; *S*_*t*_) ≤ *H*(*S*_*t*_) ≤ log|*S*| (see (Cover and Thomas, 2006)). This leads to the two following IRFs:

(8) $$R_{\text{in}}^{\text{PI}}:=|I(S_{t+1};S_{t})|\text{ and }R_{\text{in}}^{\text{H}}:=|H(S_{t})|.$$

The overall reward functions are then given by

(9) $$\begin{array}{l} R^{\text{PI}}:=\sum_{t=1}^{T}R_{\text{ex}}(t)+\beta (\gamma )R_{\text{in}}^{\text{PI}},\\ R^{\text{H}}:=\sum_{t=1}^{T}R_{\text{ex}}(t)+\beta (\gamma )R_{\text{in}}^{\text{H}},\beta (\gamma )=\gamma \cdot T\cdot \underset{x,\vartheta ,t}{\max}\left\{R_{\text{ex}}(t)\right\} \end{array}$$

where β(γ) is a factor to scale the IRF with respect to the maximal possible value of the ERF. This allows us to compare the effects of *R*^PI^_in_ and *R*^H^_in_ across different experiments.

**Figure 3:**
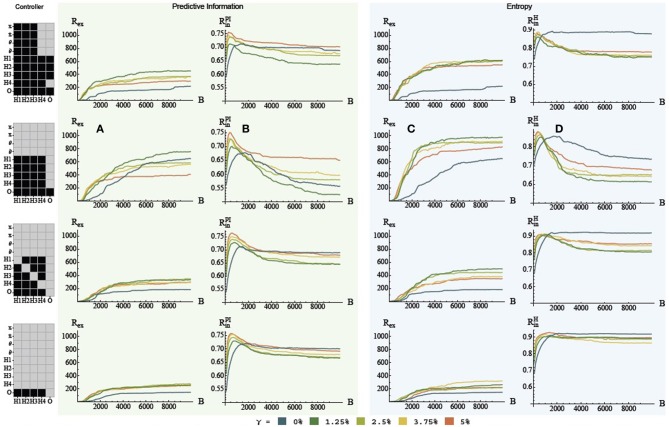
**Results for cart-pole experiments**. Each row shows the results for one controller architecture, see Figure 2. The corresponding connection matrix is provided in the first column (gray: connection, black: no connection). For simplicity only the row for the second controller is discussed in detail. **(A,B)** ERF and IRF for PI maximization—small values of γ > 0 are advantageous. **(C,D)** ERF and IRF for entropy maximization—all values of γ > 0 have positive effect.

The results are discussed only for the fully connect feed-forward network (see Figures 3A–D) in detail as this controller shows the most distinguishable results with respect to the variation of the IRF scaling parameter γ ∈ {0, 1.25, 2.5, 3.75, *and* 5%}. It is important to note that the plots only show the averages of the 100 experiments and not the standard deviation for the following reason. Few controllers succeed early, others later during the process. Due to the unsteady ERF the resulting standard deviation is very large, as those controllers that succeed receive significantly higher reward compared to those not succeeding (which remain close to zero, as a rotational behavior is not permitted). We intentionally chose an unsteady ERF, that returns zero for almost all states, and hence, we know beforehand that the standard deviation is large and no further information is provided if it is plotted.

Figures 3A,B show the progress of the ERF *R*^PI^_ex_ and IRF *R*^PI^_in_ for the PI maximization. It is shown that there is a significant speed-up in learning during the first 4000 batches for all γ > 0% (see Figure 3A). At this point in time the average ERF of γ = 0% succeeds that of γ = 5%. After approximately 5000 batches the ERF for γ = 2.5% and γ = 3.75% are very close to or slightly succeeded by the ERF for γ = 0%, whereas the ERF for γ = 1.25% remains higher. The conclusion from this experiment is that small values of γ < 5% are beneficial in this learning task as less batches are required to solve this task and the asymptotic learning performances are almost identical to γ = 0%. The results, however, are not significant and the choice of γ is critical. This leads to the conclusion that the one-step PI is not significantly beneficial in the learning of this task.

Figures 3C,D show the progress of the ERF *R*^H^_ex_ and IRF *R*^H^_in_ for the entropy maximization. The results show a different picture. Any parameter γ > 0% speeds up the learning and improves the overall performance. The comparison of entropy and PI is addressed in the discussion again.

### 3.2. Hexapod locomotion

If a specific task should be learned by an embodied agent, it is more common to choose an environment, morphology, control structure and a smooth ERF which are well-suited for the desired task. In order to investigate which effect the PI has on such a well-defined learning task, the set-up of the experiment presented in this section is chosen such that all components are known to work well if there is no IRF present. The goal is to learn a locomotion behavior of a hexapod, where the maximal deviation angles ensure that it cannot flip over. The controller is known to perform well in a similar task (Markelić and Zahedi, 2007) and its modularity significantly reduces the number of parameters that must be learned. The ERF defines a smooth gradient in the reward space, ensuring that small changes in the controller parameters show an immediate effect in the ERF. The environment is an even plane without any obstacles.

**Figure 4:**
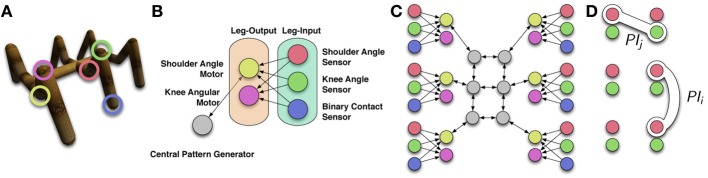
**Hexapod for locomotion task and controller set-up. (A)** Hexapod robot with marked actuated joints and sensors; **(B)** leg module of controller; **(C)** entire controller; and **(D)** schematic pairings for PI and entropy calculation.

The experimental platform (see Figure 4) is a hexapod, with 12 degrees of freedom (two actuators in each leg) and with 18 sensors (angular positions of the actuators and binary foot contact sensors). The two actuators of each leg are positioned in the shoulder (Thorax-Coxa or ThC joint) and in the knee (Femur-Tibia or FTi joint) of the walking machine, similar to the morphology presented in (von Twickel et al., 2011). We omit the second shoulder-joint (CTr) because it is not required for locomotion. Each joint accepts the desired angular position as its input and returns the actual current angular position as its output. The simulator YARS (Zahedi et al., 2008) was used for all experiments conducted in this section.

Different values for the PGPE parameters were evaluated. The best results for γ = 0 (see Equation 9) were achieved with σ_init_ = 2 and α = 0.1. To ensure comparability with the previous experiment, two roll-outs were chosen here, although it is not required to obtain the following results. The evaluation time was set to *T* = 1000 and *B* = 250 batches were sufficient to observe a convergence of the policy parameters μ. The values for γ were chosen from the previous experiment.

The ERF is calculated once at the end of each episode and it is defined as the euclidean distance between the hexapod at time *T* and its initial position (0, 0) projected onto the *xy*-plane:

(10) $$R_{\text{ex}}:=\sqrt{x_{T}^{2}+y_{T}^{2}},$$

where (*x*_*T*_, *y*_*T*_) are the coordinates of the center of the robot in world coordinates at time *t* = *T*.

The IRF is calculated differently compared to the previous experiment. In a high-dimensional system as the hexapod, it is not possible to compute the PI of the entire system with a reasonable effort, as the computational cost of *I*(*S*_*t*_; *S*_*t* + 1_) grows exponentially for every new sensor. It would be natural to reduce the computational cost by calculating the PI based on a model of the morphology, but this would violate our claim that the PI incorporates the morphology without the need of explicitly modeling it. Hence, we decided to use the following method to approximate the PI and the entropy *H* (see Figure 4D). Let *S*_*i*_(*t*), *i* = 1, 2, …, 12, be the angular position sensors for the 12 actuators. We then chose two sensors *k, l* with 1 ≤ *k, l* ≤ 12, *k* ≠ *l*, randomly from the 12 possibles sensors, and calculated

(11) $$\begin{array}{l} PI_{u}:=I\left(S_{k}(t+1),S_{l}(t+1);S_{k}(t),S_{l}(t)\right)\\ H_{u}:=H\left(S_{k}(t),S_{l}(t)\right). \end{array}$$

The overall PI and entropy are then calculated as the sum of *n* randomly chosen *PI*_*u*_ and *H*_*u*_ pairings, with the additional constraint that each sensor pair *k, l* appears only once in the approximations. The resulting IRFs are then given by:

(12) $$R_{\text{in}}^{\text{PI}}:=\sum_{u=1}^{n}PI_{u}\text{ and }R_{\text{in}}^{\text{H}}:=\sum_{u=1}^{n}H_{u},$$

where *n* is the number of pairings. For *n* > 20 no difference was found for the approximated PI, which is why *n* = 20 was chosen for the remainder of this work.

The overall reward functions are then given by:

(13) $$R^{\text{PI}}:=R_{\text{ex}}+\beta (\gamma )R_{\text{in}}^{\text{PI}}R^{\text{H}}:=R_{\text{ex}}+\beta (\gamma )R_{\text{in}}^{\text{H}}$$

where β(γ) is defined as in the cart-pole swing-up experiment (see Equation 9).

A common recurrent neural network central pattern generator layout is chosen, which can also be found in literature (e.g., Campos et al., 2010; von Twickel et al., 2011; Markelić and Zahedi, 2007), thereby using the same neuron model as in the cart-pole experiment (see above). As all legs in the hexapod are morphologically equivalent, only the synaptic weights of one leg controller are open to parameter adaptation in the PGPE algorithm. The values are then copied to the other leg controllers. This reduces the number of parameters for the entire controller to 32 (see Figures 4B,C).

**Figure 5:**
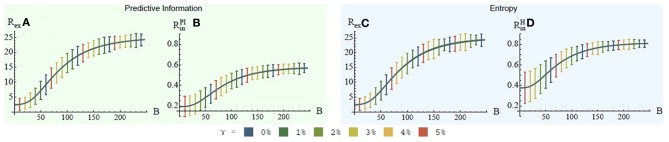
**Results for hexapod locomotion task**. ERF and IRF with PI maximization **(A,B)** and entropy maximization **(C,D)**. No significant effect is observed.

The results (see Figure 5) show that neither the PI nor the entropy have a noticeable effect on the learning performance. The mean values of the 100 experiments for each parameter as well as the standard deviation are almost identical. This point will be addressed in the discussion of this work (see Section 4).

### 3.3. Hexapod self-rescue

The third experiment is designed to combine and extend the two previous experiments. It combines them as a high-dimensional morphology, similar to that used in the locomotion experiment, is trained with an unsteady ERF, which is similar to that used in the cart-pole experiment. It extends the previous experiments as the number of parameters in the controller is a magnitude larger and because an unconventional control structure is used for the desired task. The most distinctive difference to the previous experiments is the non-trivial environment. The next paragraphs will explain the experimental set-up in detail before the section closes with a discussion of the results.

**Figure 6:**
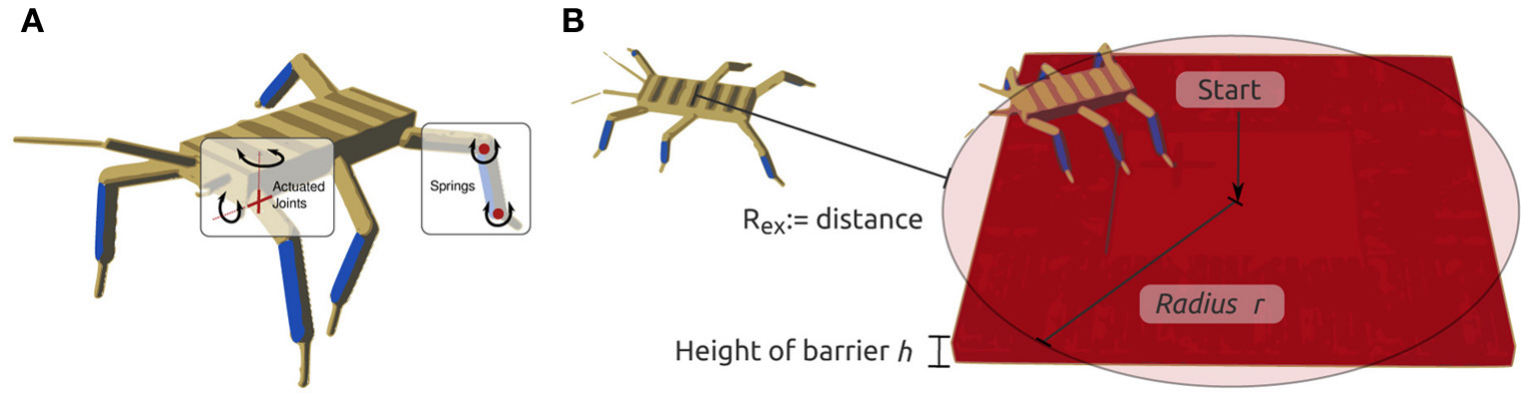
**Hexapod robot for self-rescue and the experimental setup. (A)** The robot has 6 legs where the hind legs are 10% larger than the other legs. Each leg has two active DoF in the hip joint and one passive DoF in both the knee and the ankle joint equipped with a spring. Additionally the whiskers have each two spring-joints. **(B)** The robot starts in the center of the trap with a certain barrier height and has to escape from it. The reward is the distance from the outside of the trap or zero otherwise.

We used the simulated hexapod robot of the LpzRobots simulator (Martius et al., 2012). The hexapod has 12 active and 16 passive degrees of freedom (see Figure 6). The active joints take the desired next angular position as their input and deliver the current actual angular position as their output. The controller is a fully connected one-layer feed-forward neural network without lateral connections and the hyperbolic transfer function *a*_*t* + 1_ = tanh(*Ws*_*t*_ + *v*), where *a*_*t* + 1_ and *s*_*t*_ are the next action and the current sensor values, *W* is the connection matrix, and *v* is the vector of biases. The resulting controller is parameterized by 156 parameters, 144 for the synaptic weights and 12 for the bias values. Note, that the controller is generic and has no a priori structuring or other robot-specific details.

**Figure 7:**
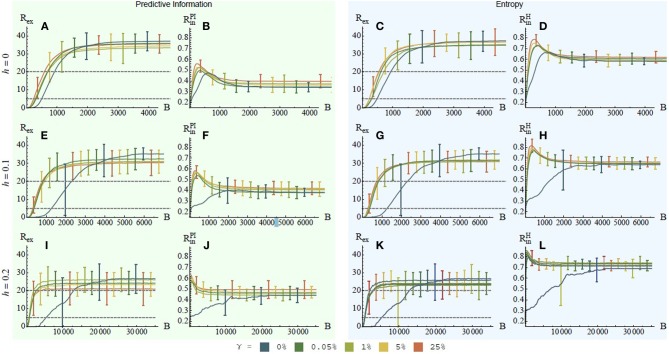
**Performance in the self-rescue task depending on the internal reward type and factor γ**. Plotted are the ERF and the IRF in case of PI **(A,B,E,F,I,J**) and entropy **(C,D,G,H,K,L**) over the number of batches for different values of γ and barrier heights *h*: **(A–D)** no barrier (*h* = 0), **(E–H)** low barrier (*h* = 0.1) and **(I–L)** high barrier (*h* = 0.2). For each value of γ the mean and standard deviation of 30 experiments are displayed. In all cases a speed-up in learning is achieved with IRF, however, the asymptotic performance is worse.

The task for the hexapod is to rescue itself from a trap. For this purpose, it is placed in a closed rectangular arena (see Figure 7). The difficulty of the task is determined by the height of the arena's walls, denoted by *h* ∈ {0.0m, 0.1m, 0.2m} (see Figure 6). For comparison, the length of the lower leg (up to the knees) is 0.45 m. The size-proportion of the robot and the trap can be seen in Figure 6B.

The ERF is given by

(14) $$R_{\text{ex}}\;:=\{\begin{array}{ll} \sqrt{x_{T}^{2}+y_{T}^{2}}-r & \text{if }\sqrt{x_{T}^{2}+y_{T}^{2}}-r>0\\ 0 & \text{otherwise}, \end{array}$$

where *r* is the radius of the trap (Figure 6) and (*x*_*T*_, *y*_*T*_) is the position of the center of the robot in world coordinates at the end of a roll-out (*t* = *T*). The IRFs and overall reward functions are identical to those used in the previous experiment (see Equations (11) and (12)).

As before, the performance of PGPE with γ = 0 for different values for σ_init_ and α were evaluated, and the best are chosen for presentation here, which are σ_init_ = 2 and α = 0.5. A different learning rate α_σ_ = 0.05 was chosen for the update of σ (see Equation 3). Each episode consisted of *T* = 1250 iterations (25s) with one roll-out per episode. A total of *B* = 5000, 7000, and 35000 batches were conducted for the different heights *h*.

We compare the performance for different values of the IRF factor γ ∈ {0, 0.05, 1, 5, and 25%} and performed 30 experiments for each setting. Figure 7 displays the results. As for the cart-pole experiment, the plots for the PI and entropy in Figure 7 report a clear picture of an exploration phase (high value) followed by an exploitation phase (lower value).

To compare the results, we set two threshold values at *R*_ex_ = 5 and *R*_ex_ = 20 which refer to a 5m and 20m distance between the hexapod and the walls of the arena. The first threshold reflects a successful learning of the task, because it means that hexapod reliably escapes the arena. The second threshold represents the case when in addition also a high locomotion speed is achieved after a successful escape. For the simplicity of argumentation, we compare two cases, i.e., γ = 0% and γ = 1%. If there is no wall (*h* = 0m) the system with IRF γ = 1% requires only half the amount of batches compared to no IRF (250 batches vs. 500 batches, see Figures 7A,C). For the arena with a medium height (*h* = 0.1m), the learning success speed ratio increases to approximately three (350 batches vs. 1100 batches, see Figures 7E,F). The results are decisive for the arena with high walls (*h* = 0.2m), as the system with IRF requires about 1000 batches on average compared to the 5000 batches on average that a required by the systems without IRF (see Figures 7I,K).

This leads to the conclusion that both, PI and entropy, are beneficial if the short-term learning success is of the primary interest. However, the asymptotic learning success of those hexapods with IRF is either equal or lower compared to those without an IRF in all experiments. This is valid for the one-step PI and for the entropy. Thus, both are not necessarily beneficial if the long-term, asymptotic learning performance in an episodic policy gradient setting is important.

## 4. Discussion

This paper discussed the one-step PI (Bialek et al., 2001) as an information-driven intrinsic reward in the context of an episodic policy gradient method. The reward is considered to be intrinsic, because it is task-independent and it relies only on the information of the sensors of an agent, which, by definition, represent the agent's intrinsic view on the world. We chose the maximization of the one-step PI as an IRF, because it has proved to encourage behaviors which show properties of morphological computation without the need to model the morphology (Zahedi et al., 2010).

The IRF was linearly combined with a task-dependent ERF in an episodic RL setting. Specifically, PGPE (Sehnke et al., 2010) was chosen as RL method, because it allows to learn arbitrary policy parametrizations. Within this set-up, three different types of experiments were performed. The following paragraph will summarize the results before they are discussed.

The first experiment was the learning of the cart-pole swing-up task. Four controllers were evaluated of which three were less successful and one showed good results. The ERF was designed to be difficult to maximize without the IRF, and the task contradicted the maximization of the entropy and PI. The best controller did not show a significant improvement of the learning performance with respect to its asymptotic behavior. An improvement could only be observed during the first learning steps. Moreover, the choice of the linear combination factor γ is critical. For all controllers a minor and not significant improvement is observable. In case of the entropy maximization, any factor γ > 0% showed an improvement in learning speed and learning performance.

A locomotion behavior was learned for a hexapod in the second experiment. The entire set-up used well-known components for the environment, modular controller, ERF, and morphology so that the task was solved without IRF in only a few learning steps. No effect of the PI and entropy was observed.

The third experiment combined the previous two and extended them by a non-trivial environment. A hexapod had to escape from a trap and was only rewarded outside of it. The results showed no significant difference between the PI and the entropy as IRFs. The learning speed was significantly improved by both IRFs with increasing difficulty of the task. The asymptotic performance was either equal or worse when an IRF was introduced.

The hexapod locomotion experiment teaches us that the information-theoretic reward functions (PI and entropy) has no effect in well-defined experimental set-ups.

The cart-pole and the hexapod self-rescue experiments teach us that the maximal values of the IRF should be around one percent of the maximal ERF value to improve the learning speed and learning performance in the short-term. The asymptotic behavior is either not or negatively effected by the one-step PI. The cart-pole experiment indicates that maximizing the entropy is superior to maximizing the PI, whereas the hexapod self-rescue does not show such a clear picture. The success of the entropy in both experiments is explained by the ERFs. Due to their nature, random changes in the policy parameters are unlikely to result in changes in the ERF during the first batches. Hence, maximizing the entropy results in an exploration until the ERF is triggered.

The PI, defined as the entropy over the sensor states subtracted by the conditional entropy of consecutive sensor states does not result in superior results for the cart-pole compared to just using the entropy for the following reason. In this set-up, the morphology and environment are very simple and deterministic, and therefore, do not produce any noise or other uncertainties in the sensor data stream. The uncertainty about the next possible angular position of the pole is small, if the current pole position is known. In other words, the cart-pole system is regular by definition and no further regularities can be found by maximizing the PI. We speculate that the conditional entropy, which cannot be reduced by the learning in this setting, dampens the exploration effect of the entropy term in the PI maximization. For the hexapod rescue experiment, the situation is different. There is an uncertainty about the next sensor state, given the current sensor state which result from the morphology and the construction of the arena. The PI maximization is able to find regularities which can then be exploited to maximize the ERF in the RL setting.

The results contradict our intuition, as the one-step predictive information has shown good results when applied as an information-driven self-organization principle in the context of embodied artificial intelligence (Zahedi et al., 2010; Martius et al., 2013). The intuitively plausible next step was to guide the information-driven self-organization toward solving a goal by combining it with an external reward signal in an reinforcement learning context. The approach evaluated in this paper was to linearly combine the PI with and external reward signal in an episodic policy gradient learning. If anything, then the PI showed positive short-term results, if the world was considerably probabilistic and if the external reward was sparse. Compared to no intrinsic reward the PI showed negative results for its asymptotic behavior. The performance of the PI was either equal or worse compared to the entropy in all cases. This leads to the conclusion that research in the context of information-driven intrinsic rewards and reinforcement learning should be carried out in other directions, which are briefly described in the final paragraph.

We have used a constant combination factor γ for all experiments presented in this work. It is known from general learning theory that a decaying learning rate is required for the convergence of a system. We chose not to use a decaying learning factor, because this means that the internal drive is slowly dampened until its effect is neglectable (at least in a technical application). This would contradict the idea of motivation-driven and open-ended learning of embodied agents. However, the results of our present paper reveal a disadvantage of this approach in the asymptotic limit, and therefore, suggest, contrary to our original thoughts, to pursue a strategy with a decaying combination factor. The second possible modification of this approach is to exchange the linear combination of the internal and external reward by a non-linear function, of which multiplicative and exponential functions are two examples. Third, using a gradient of the PI instead of a random exploration in the context of RL is a promising approach that is currently investigated. In this approach, we will use a gradient on an estimate of the PI and not the error of a predictor as in e.g., (Schmidhuber, 1991). Fourth, we will continue to evaluate other information-theoretic measures in the context of task-dependent learning with the support of information-driven intrinsic motivation. In addition to using correlation measures, such as the mutual information, we believe that causal measures in the sensorimotor loop (Ay and Zahedi, 2013), such as the measure considered in (Zahedi and Ay, 2013), are good candidates for future research in this field.

### Conflict of interest statement

The authors declare that the research was conducted in the absence of any commercial or financial relationships that could be construed as a potential conflict of interest.

## References

[B1] Ay N. Polani D. (2008). Information flows in causal networks. Adv. Complex Syst. 11, 17–41 10.1142/S0219525908001465

[B2] Ay N. Zahedi K. (2013). An information theoretic approach to intention and deliberative decision-making of embodied systems, in Advances in Cognitive neurodynamics III ed Yamaguchi Y. (Heidelberg: Springer).

[B3] Ay N. Bertschinger N. Der R. Güttler F. Olbrich E. (2008). Predictive information and explorative behavior of autonomous robots. Eur. Phys. J. B 63, 329–339 10.1140/epjb/e2008-00175-0

[B4] Barto A. G. Singh S. Chentanez N. (2004). Intrinsically motivated learning of hierarchical collections of skills, in Proceedings of 3rd International Conference on Developmental Learning, (San Diego, CA), 112–119

[B5] Barto A. G. Sutton R. S. Anderson C. W. (1983). Neuron like adaptive elements that can solve difficult learning control problems. IEEE Trans. Syst. Man. Cybern. SMC-13, 834–846 10.1109/TSMC.1983.6313077

[B6] Bellman R. E. (2003). Dynamic Programming. Mineola, NY: Dover Publications, Incorporated

[B7] Bialek W. Nemenman I. Tishby N. (2001). Predictability, complexity, and learning. Neural Comput. 13, 2409–2463 10.1162/089976601753195969 11674845

[B8] Brooks R. A. (1991). Intelligence without reason, in Proceedings of the 12th International Joint Conference on Artificial Intelligence (IJCAI-91), eds Myopoulos J. Reiter R. (San Mateo, Sydney: Morgan Kaufmann publishers Inc.), 569–595

[B9] Campos R. Matos V. Santos C. (2010). Hexapod locomotion: a nonlinear dynamical systems approach, in Conference Of IEEE Industrial Electronics. Proceedings, (Glendale, AZ), 1546–1551

[B10] Clark A. (1996). Being There: Putting Brain, Body, and World Together Again. Cambridge, MA: MIT Press

[B11] Cover T. M. Thomas J. A. (2006). Elements of Information Theory, Vol. 2 Hoboken, NJ: Wiley

[B12] Crutchfield J. P. Young K. (1989). Inferring statistical complexity. Phys. Rev. Lett. 63, 105–108 10.1103/PhysRevLett.63.105 10040781

[B13] Cuccu G. Luciw M. Schmidhuber J. Gomez F. (2011). Intrinsically motivated evolutionary search for vision-based reinforcement learning, in Proceedings of the 2011 IEEE Conference on Development and Learning and Epigenetic Robotics IEEE-ICDL-EPIROB, (Frankfurt: IEEE).

[B14] Dayan P. Balleine B. W. (2002). Reward, motivation, and reinforcement learning. Neuron 36, 285–298 10.1016/S0896-6273(02)00963-7 12383782

[B15] Der R. Martius G. (2012). The Playful Machine: Theoretical Foundation and Practical Realization of Self-Organizing Robots (Cognitive Systems Monographs). Berlin; Heidelberg: Springer

[B16] Doya K. (2000). Reinforcement learning in continuous time and space. Neural Comput. 12, 219–245 10.1162/089976600300015961 10636940

[B17] Geva S. Sitte J. (1993). The cart pole experiment as a benchmark for trainable controllers. IEEE Control Syst. Mag. 13, 40–51 10.1109/37.236324

[B18] Grassberger P. (1986). Toward a quantitative theory of self-generated complexity. Int. J. Theor. Phys. 25, 907–938 10.1007/BF00668821

[B19] Kaplan F. Oudeyer P.-Y. (2004). Maximizing learning progress: an internal reward system for development, in Embodied Artificial Intelligence, eds Iida F. Pfeifer R. Steels L. Kuniyoshi Y. (Berlin; Heidelberg: Springer-Verlag), 259–270

[B20] Klyubin A. S. Polani D. Nehaniv C. L. (2004). Organization of the information flow in the perception-action loop of evolved agents, in Proceedings of the 2004 NASA/DoD Conference on Evolvable Hardware, 2004, (Seattle, WA), 177–180

[B21] Little D. Y. Sommer F. T. (2013). Learning and exploration in action-perception loops. Front. Neural Circuits 7:37 10.3389/fncir.2013.00037 23579347 PMC3619626

[B22] Markelić I. Zahedi K. (2007). An evolved neural network for fast quadrupedal locomotion, in Advances in Climbing and Walking Robots, Proceedings of 10th International Conference (CLAWAR 2007), eds Xie M. Dubowsky S. (World Scientific Publishing Company), 65–72

[B23] Martius G. Der R. Ay N. (2013). Information driven self-organization of complex robotic behaviors. PLoS ONE Singapore 8:e63400 10.1371/journal.pone.0063400 23723979 PMC3664628

[B24] Martius G. Herrmann J. M. (2012). Variants of guided self-organization for robot control. Theory Biosci. 131, 129–137 10.1007/s12064-011-0141-0 22116785

[B25] Martius G. Hesse F. Güttler F. Der R. (2012). LpzRobots: a free and powerful robot simulator, version 0.7. Available online at: http://robot.informatik.uni-leipzig.de/software

[B26] Oudeyer P.-Y. Kaplan F. Hafner V. V. (2007). Intrinsic motivation systems for autonomous mental development. IEEE Trans. Evo. Comput. 11, 265–286 10.1109/TEVC.2006.890271

[B27] Pasemann F. Steinmetz U. Dieckman U. (1999). Evolving structure and function of neurocontrollers, in Proceedings of the Congress Evolutionary Computation CEC 99, Vol. 3, (Washington, DC).

[B28] Pfeifer R. Bongard J. C. (2006). How the Body Shapes the Way We Think: A New View of Intelligence, (Cambridge, MA: The MIT Press; Bradford Books).

[B29] Pfeifer R. Lungarella M. Iida F. (2007). Self-organization, embodiment, and biologically inspired robotics. Science 318, 1088–1093 10.1126/science.1145803 18006736

[B30] Prokopenko M. Gerasimov V. Tanev I. (2006). Evolving spatiotemporal coordination in a modular robotic system, in Proceedings on SAB'06, Vol. 4095, (Rome, Italy), 558–569

[B31] Schmidhuber J. (1990). A possibility for implementing curiosity and boredom in model-building neural controllers, in Proceedings of SAB'90, (Cambridge, MA), 222–227

[B32] Schmidhuber J. (1991). Curious model-building control systems, in In Proceedings on International Joint Conference on Neural Networks, Singapore, (IEEE), 1458–1463

[B33] Schmidhuber J. (2006). Developmental robotics, optimal artificial curiosity, creativity, music, and the fine arts. Connect. Sci. 18, 173–187 10.1080/09540090600768658

[B34] Sehnke F. Osendorfer C. Rückstiess T. Graves A. Peters J. Schmidhuber J. (2010). Parameter-exploring policy gradients. Neural Netw. 23, 551–559 10.1016/j.neunet.2009.12.004 20061118

[B35] Storck J. Hochreiter S. Schmidhuber J. (1995). Reinforcement driven information acquisition in non-deterministic environments, in Proceedings of the International Conference on Artificial Neural Networks, Vol. 2, (Paris: EC2 & Cie), 159–164

[B36] Sutton R. S. Barto A. G. (1998). Reinforcement Learning: An Introduction, Cambridge, MA: MIT Press

[B37] von Twickel A. Büschges A. Pasemann F. (2011). Deriving neural network controllers from neuro-biological data: implementation of a single-leg stick insect controller. Biol. Cybern. 104, 95–119 10.1007/s00422-011-0422-1 21327828

[B38] von Uexkuell J. (1934). A stroll through the worlds of animals and men, in Instinctive Behavior, ed Schiller C. H. (New York, NY: International Universities Press), 5–80

[B39] Yi S. Gomez F. Schmidhuber J. (2011). Planning to be surprised: optimal Bayesian exploration in dynamic environments, in Proceedings on Fourth Conference on Artificial General Intelligence (AGI), (Mountain View, CA: Google).

[B40] Zahedi K. Ay N. (2013). Quantifying morphological computation. Entropy 15, 1887–1915 10.3390/e15051887

[B41] Zahedi K. Ay N. Der R. (2010). Higher coordination with less control—a result of information maximization in the sensori-motor loop. Adapt. Behav. 18, 338–355 10.1177/1059712310375314

[B42] Zahedi K. von Twickel A. Pasemann F. (2008). Yars: a physical 3d simulator for evolving controllers for real robots, in SIMPAR 2008 Vol. 5325, eds Carpin S. Noda I. Pagello E. Reggiani M. von Stryk O. (Berlin; Heidelberg: Springer), 71—82

